# Probucol mitigates high-fat diet-induced cognitive and social impairments by regulating brain redox and insulin resistance

**DOI:** 10.3389/fnins.2024.1368552

**Published:** 2024-04-23

**Authors:** Han-Ming Wu, Yang Vivian Yang, Na-Jun Huang, Li-Ping Fan, Ying-Ying Dai, Ke-Ting Hu, Tian-Yu Tang, Lin Liu, Yue Xu, Dong-Tai Liu, Ze-Xin Cai, Xiao-Yu Niu, Xin-Yi Ren, Zheng-Hao Yao, Hao-Yu Qin, Jian-Zhen Chen, Xi Huang, Cixiong Zhang, Xiang You, Chen Wang, Ying He, Wei Hong, Yu-Xia Sun, Yi-Hong Zhan, Shu-Yong Lin

**Affiliations:** ^1^Department of Neurology, Xiang'an Hospital of Xiamen University, School of Medicine, Xiamen University, Xiamen, China; ^2^Department of Neurology and Department of Neuroscience, The First Affiliated Hospital of Xiamen University, School of Medicine, Xiamen University, Xiamen, China; ^3^State Key Laboratory of Cellular Stress Biology, School of Life Sciences, Faculty of Medicine and Life Sciences, Xiamen University, Xiamen, China; ^4^The Third Clinical Medical College, Fujian Medical University, Fuzhou, China; ^5^School of Medicine, Faculty of Medicine and Life Sciences, Xiamen University, Xiamen, China; ^6^Laboratory Animal Center, Xiamen University, Xiamen, China; ^7^Institute of Metabolism and Health, Henan University, Zhengzhou, China; ^8^Department of Digestive Diseases, School of Medicine, Faculty of Medicine and Life Sciences, Xiamen University, Xiamen, China

**Keywords:** probucol, high-fat diet, spatial cognition, social behavior, redox homeostasis, insulin resistance

## Abstract

Probucol has been utilized as a cholesterol-lowering drug with antioxidative properties. However, the impact and fundamental mechanisms of probucol in obesity-related cognitive decline are unclear. In this study, male C57BL/6J mice were allocated to a normal chow diet (NCD) group or a high-fat diet (HFD) group, followed by administration of probucol to half of the mice on the HFD regimen. Subsequently, the mice were subjected to a series of behavioral assessments, alongside the measurement of metabolic and redox parameters. Notably, probucol treatment effectively alleviates cognitive and social impairments induced by HFD in mice, while exhibiting no discernible influence on mood-related behaviors. Notably, the beneficial effects of probucol arise independently of rectifying obesity or restoring systemic glucose and lipid homeostasis, as evidenced by the lack of changes in body weight, serum cholesterol levels, blood glucose, hyperinsulinemia, systemic insulin resistance, and oxidative stress. Instead, probucol could regulate the levels of nitric oxide and superoxide-generating proteins, and it could specifically alleviate HFD-induced hippocampal insulin resistance. These findings shed light on the potential role of probucol in modulating obesity-related cognitive decline and urge reevaluation of the underlying mechanisms by which probucol exerts its beneficial effects.

## Introduction

Obesity and its negative metabolic consequences such as type 2 diabetes and hyperlipidemia, are generally regarded as conferring risk factors for impairments in the central nervous system (CNS) (Vinik et al., 2000; McCrimmon et al., 2012; Kiliaan et al., 2014; Fulton et al., 2022; Kouvari et al., 2022). Consumption of calorie-dense diets, which is considered as one of the major factors contributing to the obesity pandemic, has been linked to cognitive dysfunction and mood-related behaviors in humans (Ortega et al., 1997; Morris et al., 2003; Kalmijn et al., 2004; Morris et al., 2004; Eskelinen et al., 2008; Gibson et al., 2013; Baker et al., 2017). Numerous animal studies demonstrated that high-fat diet (HFD) feeding, which is routinely utilized to establish obese animal models, leads to impaired learning and memory, and induces anxiety- and depression-like behaviors (Moon et al., 2014; Zemdegs et al., 2016; Abbott et al., 2019; Han et al., 2020; Seguella et al., 2021; Yu et al., 2021; Zhuang et al., 2022). Insulin resistance, altered lipid homeostasis, increased systemic oxidative stress and chronic inflammation, as well as dysfunctional vascularization under obese conditions may promote the development of structural and molecular changes in the brain (Greenwood and Winocur, 1990; Freeman et al., 2014; Beilharz et al., 2015; Schachter et al., 2018; Huang Q. et al., 2019; Sharma, 2021).

Various strategies, such as nutrition, anti-obesity drugs and exercise, have been tested to assess their effects on mitigating cognitive and mood-related impairments induced by HFD (Park et al., 2019; Ruegsegger et al., 2019; Nuzzo et al., 2020; Wang et al., 2021). Expectedly, most of these treatments demonstrated improvement in obesity-related metabolic abnormalities, including reduction of body weight, fat content, serum glucose level, and insulin resistance to varying extents, alongside their diverse influences on the CNS. However, several studies indicated that the beneficial effects on cognitive decline and anxiety can be achieved without directly targeting the systemic metabolic changes induced by HFD (Chou et al., 2016; Jaiswal et al., 2018; Sona et al., 2018; Ogrodnik et al., 2019; Shi et al., 2020; Wang et al., 2020; Wu et al., 2021). These findings suggest the presence of multiple pathways that mediate the impact of dietary intake on specific CNS abnormalities.

Redox homeostasis stands out as a key area of research within these pathways. Lipid oxidation products, such as oxidized low-density lipoprotein (oxLDL) and malondialdehyde (MDA), as well as nitric oxide (NO) derived from the inducible nitric oxide synthase (iNOS), are positively correlated with obesity severity (Prazny et al., 1999; Couillard et al., 2005; Weinbrenner et al., 2006; Ctoi et al., 2018). These molecules play critical roles in neuroinflammation, triggering the production of proinflammatory cytokines, such as tumor necrosis factor-α. Moreover, certain radical species directly interact with cellular macromolecules like lipids, DNA, and proteins. Consequently, these effects contribute to cellular component damage and tissue destruction (Wei et al., 1995; Romero et al., 1998; Chamorro et al., 2016). Nonetheless, it is evident that reactive oxygen species (ROS) may also play notable roles in various physiological processes, such as brain development and plasticity, post-trauma angiogenesis, and elimination of dysfunctional cells (Angelova and Abramov, 2018; Stefanatos and Sanz, 2018; Sutkowy et al., 2021). Consistently, although several small-scale clinical or laboratory studies demonstrated the beneficial effects of antioxidants on mitigating cognitive decline and mood-related disorders (Maczurek et al., 2008; Franzoni et al., 2021; Caruso et al., 2022; Martinez-Banaclocha, 2022), large-scale clinical trials generally do not yield significant positive outcomes (Kang et al., 2009; Rautiainen et al., 2016). Hence, modulating the activity and specificity of the antioxidants may be crucial to achieve context-dependent oxidant-antioxidant balance.

Probucol, known for its utilization as a cholesterol-lowering drug, has also demonstrated antioxidant properties by inhibiting the oxidative modification of low-density lipoprotein (LDL) (Barnhart et al., 1970; Parthasarathy et al., 1986). The beneficial effects of probucol on cognitive dysfunction have been reported in pathological models, including mice injected with Amyloid beta proteins (Santos et al., 2012), those with D-galactose-induced cognitive deficits (Huang J. L. et al., 2019; Xie et al., 2021), and those with drug or high-cholesterol diet-induced diabetes (Santos et al., 2015; Mamo et al., 2019). However, clinical trials suggested that the metabolic and antioxidative effects of probucol were relatively mild and primarily found in cases with very high cholesterol levels (Yamashita et al., 2008; Cholesterol Treatment Trialists et al., 2015; Yamashita et al., 2016, 2021). It remains unclear whether the cholesterol-reducing and antioxidant effects of probucol universally underlie these positive effects. The present study aimed to explore the influences and underlying mechanisms of probucol on obesity induced-cognitive decline.

## Methods

### Animals

C57BL/6 J mice were supplied by the Laboratory Animal Center of Xiamen University (Xiamen, China). In this study, 2-month-old male mice were provided *ad libitum* access to either a standard chow diet (NCD) (D12450B, Research Diets, New Brunswick, NJ, United States) or an HFD (D12492, Research Diets), comprising 20% protein, 20% carbohydrates, and 60% fat (derived from lard and soybean oil) with no supplemental cholesterol (Supplementary Figure S1). Mice were housed in an environment maintained at 22°C, with a humidity of 55–60%, under a 12-h light/dark cycle. Nesting materials within the cages were replaced once weekly. Mice were divided into 3 groups based on their diet and pharmacological intervention. The first group, the NCD group, was fed a NCD without probucol treatment. The second group, the HFD group, consumed a HFD without probucol treatment. The third group, the HFD + probucol group, was fed the HFD starting 8 weeks prior to administration of 0.005% probucol in their drinking water for 12 weeks (11–12 mice per group). Both probucol-treated and non-treated mice were housed separately in cages, with each cage containing 5–6 mice. The consumption of water and food was monitored per cage following the 12-week probucol treatment. The animal experiments were approved by the Institutional Animal Care and Use Committee at Xiamen University (Approval No. XMULAC20180103).

### Behavioral tests

**Figure 1:**
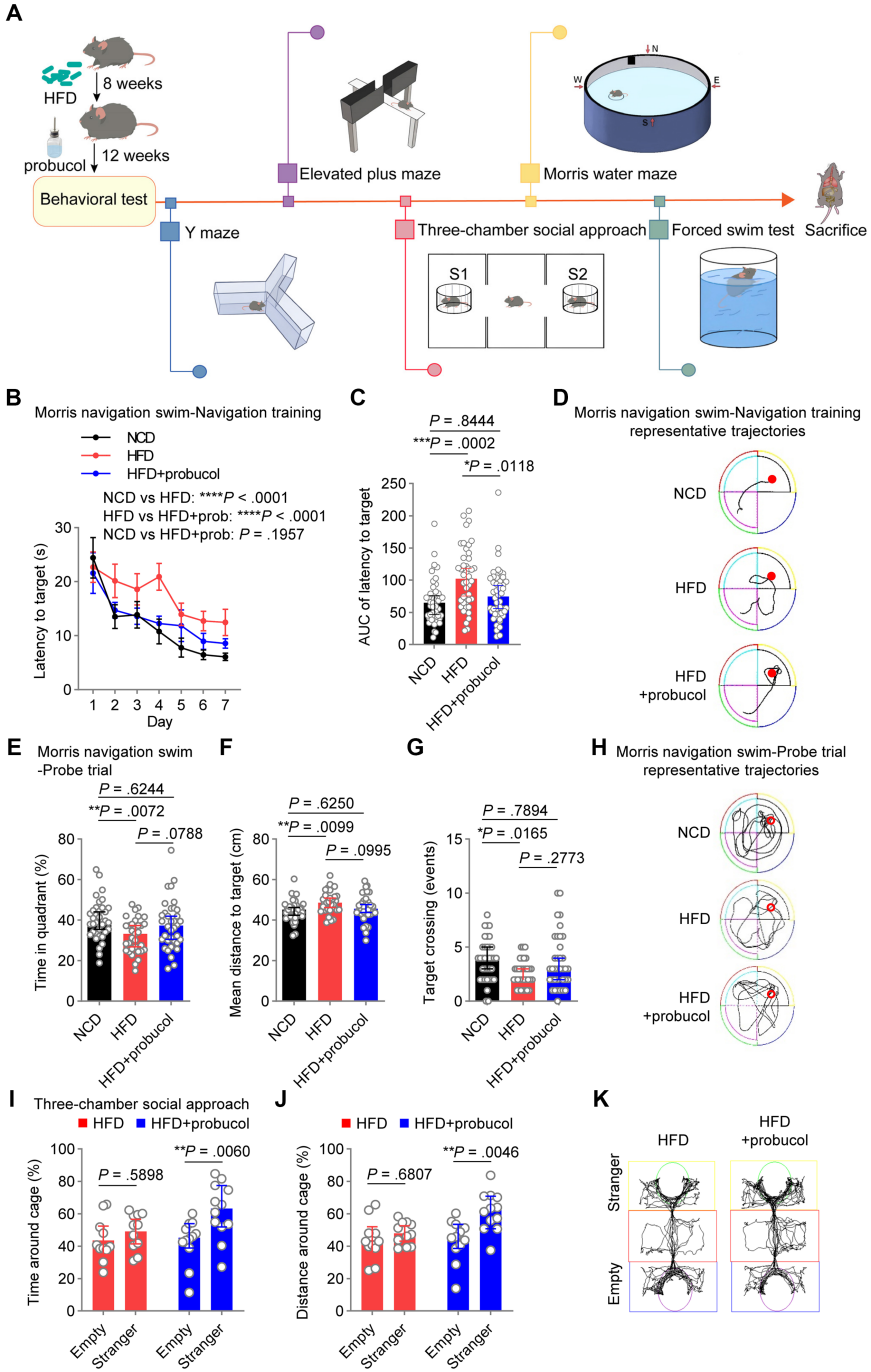
Probucol counteracts HFD-induced deficits in spatial and social cognition. **(A)** Graphic workflow of the study. C57BL/6 J mice were assigned to either the NCD group or the HFD group for 8 weeks. Subsequently, HFD-fed mice were divided into two groups. One group received drinking water supplemented with probucol, while the other HFD group and the NCD group maintained their original diet without any supplementary treatment. After 12 weeks of probucol intervention, mice were subjected to Y maze, elevated plus maze test, three-chamber social approach task, Morris water maze test, and forced swim test, sequentially. Mice were sacrificed after the behavioral tests. **(B,C)** Performance of mice in navigation training of the Morris swim navigation task. The training was stopped when more than 90% of mice found the platform within 30 s for two consecutive days. Data of latency to target are shown in **(B)** as mean ± SEM (*n* = 11 or 12 mice for each group, and 4 starting points per mouse). Ordinary two-way ANOVA (*p* < 0.0001), followed by Holm-Sidak’s multiple comparisons test. The area under the curve (AUC) of the latency to target of each mouse is illustrated in **(C)** as individual values with median ± 95% CI. Kruskal-Wallis test (*p* = 0.0003), followed by Dunn’s multiple comparisons test. **(D)** Representative trajectories of mice on day 4 of the navigation training showing latency to the platform. **(E–G)** Performance of mice in the probe trials of the Morris swim navigation task. After reaching the standard of navigation training, the probe trials were conducted on mice described in A by removing the hidden platform. Data are expressed as individual values with median ± 95% CI (*n* = 11 or 12 mice for each group, and 3 starting regions per mouse). Time in quadrant, ordinary one-way ANOVA (*p* = 0.0085), followed by Tukey’s multiple comparisons test. Mean distance to targets, ordinary one-way ANOVA (*p* = 0.0118), followed by Tukey’s multiple comparisons test. Target crossing number, Kruskal-Wallis test (*p* = 0.0204), followed by Dunn’s multiple comparisons test. **(H)** Representative full trajectories of mice in the probe trials of the Morris swim navigation task. **(I,J)** Social interaction of mice in the three-chamber social approach task. Stranger and Empty in the sociability task indicate the cage with a novel stranger male mouse and an empty cage, respectively. Data are presented as individual values with median ± 95% CI (*n* = 11 or 12 mice for each group). Time around cage, two-way repeated measure (RM) ANOVA (cage, *p* = 0.0069; group, *p* = 0.1689), followed by Sidak’s multiple comparisons test. Distance around cage, two-way RM ANOVA (cage, *p* = 0.0073; group, *p* = 0.0341), followed by Sidak’s multiple comparisons test. **(K)** Representative trajectories of HFD-fed mice and probucol treated mice in the three-chamber social approach task. *p* < 0.05 was indicative of statistical significance.

A discrete room within the animal facility, maintained at 22°C with 40–70% humidity, was designated for murine behavioral tests to minimize external disturbances. Diffuse reflection light source was employed to ensure a subdued lighting environment. Mice were acclimated to the experimenters for at least 1 week before the commencement of the experiments. Additionally, they were housed in the testing room for 2 days prior to the initial test to acclimate them to the new environment. Experimenters took precautions to prevent odorant contamination by showering and changing clothes before each test session. Sequentially, mice underwent Y maze, elevated plus maze (EPM) test, three-chamber social approach task, Morris water maze (MWM) test, and forced swim test. Silence was maintained during the tests. After each round of testing, the apparatus used was wiped with 75% ethanol to remove any residual odors from mice. The duration of each test is specifically described in the following sections. The study design is illustrated in Figure 1A.

### MWM test

The MWM test was performed as previously described with some modifications (Morris, 1984; Vorhees and Williams, 2006). The water maze comprised of a round tank with a diameter of 90 cm, filled with water that was maintained at approximately 22°C (Xiamen Baocheng Biotechnology, Xiamen, China). In each quadrant of the pool wall, graphic clues were affixed. The test was conducted from 18:00 to 20:30. Throughout the navigation training, a fixed platform was placed in one of the four quadrants. Mice underwent training for 7 days, including four sessions per day, starting from four different locations in a pseudorandom manner. Training continued until 90% of the mice could successfully locate the platform within 30 s for two consecutive days. On the eighth day, spatial memory capacity was evaluated using a spatial probe test, wherein the platform was removed. Mice were given 60 s to search for the original location of the platform. The performance of mice was recorded and analyzed using an automatic tracking system (SMARTPREMIUM 3.3, Panlab Harvard Apparatus, Barcelona, Spain). The latency to target and the area under the curve (AUC) of latency to target during acquisition were analyzed to assess spatial learning and memory capabilities of mice. The percentage of time spent on the target quadrant, mean distance to target, and total crossing number during the probe trials were also analyzed to evaluate the spatial navigation ability of mice.

### Three-chamber social approach task

The three-chamber social approach task was conducted as previously described with some modifications (Yang et al., 2011). The apparatus used was a rectangular box divided into three chambers, measuring 60 cm × 40 cm (Xiamen Baocheng, Fujian, China). The task was conducted from 11:00 to 18:00. Mice were placed in the middle chamber and given access to both end chambers, each equipped with a wired cage. During the habituation phase, cages in both end chambers were empty. In the sociability test, one stranger mouse was placed inside one of the cages, while the other cage remained empty. In the social preference test, a second stranger mouse was placed inside the wired cage in the opposite side chamber. Each session lasted for 5 min for the habituation phase, 10 min for the sociability test, and 5 min for the social preference test. The movements and interactions of mice were recorded and analyzed using an automatic tracking system (SMARTPREMIUM 3.3, Panlab Harvard Apparatus, Barcelona, Spain). The interaction region was defined as a 3 cm area surrounding the wire cage. Videos were carefully examined to assess the performance of each mouse in the social interaction test, with specific attention given to ensure that no mice exhibited climbing behavior in cages.

### EPM test

The EPM used in this study was a customized four-armed apparatus. Each arm measured 30.8 cm × 6 cm × 16 cm (Xiamen Baocheng) and was elevated 65 cm off the floor. The tasks were conducted from 17:00 to 21:00. Mice were placed in the center of the maze, facing the closed arms, and allowed to explore freely for 5 min. The movements and behaviors of mice were recorded and analyzed by an automatic tracking system (SMARTPREMIUM 3.3, Panlab Harvard Apparatus). Additionally, the head dipping behavior, which is known to decrease in response to anxiety in the EPM, was accurately assessed through manual counting.

### Forced swim test

The forced swim test utilized a cylindrical water tank with a height of 30 cm and a diameter of 15 cm (Xiamen Baocheng). The water level in the tank was maintained at approximately 15 cm above the bottom and kept at a temperature of around 22°C. The test was conducted from 17:00 to 21:30. Mice were released into the tank and allowed to freely explore for 6 min. The movements of mice were recorded and analyzed by an automatic tracking system (SMARTPREMIUM 3.3, Panlab Harvard Apparatus). Global activity is the sum of the difference between two consecutive frames of the acquired images. A mouse was considered to be in an immobile state when it had been continuously rested for 0.5 s. Extended periods of immobility are indicative of greater expression of depressive-like behaviors.

### Y maze test

The Y maze used in this study was a customized apparatus with three arms (37.8 cm × 6.7 cm × 16 cm arms, Xiamen Baocheng). The test was conducted from 15:30 to 21:00. Mice were placed in the center of the Y maze, facing the direction of one of the arms. They were allowed to freely explore the maze for 8 min. The movements of mice were recorded and analyzed using an automatic tracking system (SMARTPREMIUM 3.3, Panlab Harvard Apparatus). The alternation triplet represents the count of three consecutive entries made into different arms of the maze. It is calculated as a percentage using the following formula: Alternation triplet (%) = Number of alternation triplets / (total arm entries - 2) × 100. A lower percentage of alternative triplet is indicative of impairment in spatial working memory.

### Immunoblotting

The cerebral cortex and hippocampus from euthanized mice were extracted from the left cerebral hemisphere and briefly washed in ice-cold phosphate-buffered saline (PBS). Afterwards, the collected tissues were subjected to homogenization and sonication in radioimmunoprecipitation assay buffer (1 × PBS, 1% NP-40, 0.1% sodium dodecyl-sulfate (SDS), 0.6% sodium deoxycholate, and phosphatase and protease inhibitor cocktails). The homogenates were subsequently centrifuged at 20000 g for 15 min. The supernatants were collected and the protein concentrations were determined, ensuring equal loading of 20 μg protein for each sample, which were then subjected to SDS-polyacrylamide gel electrophoresis (SDS-PAGE) and electrophoretic transfer. Immunoblotting was performed following the protocols provided by the primary antibody manufacturers. In this study, the following primary antibodies were utilized for immunoblotting: iNOS polyclonal Antibody (1:1000, 18,985-1-AP, Proteintech, Wuhan, China), nicotinamide adenine dinucleotide phosphate oxidase 2 (NOX2) polyclonal antibody (1:5000, 19,013-1-AP, Proteintech), phospho-AKT (Ser473) polyclonal antibody (1:1000, 28,731-1-AP, Proteintech), AKT polyclonal antibody (1:2000, 10,176-2-AP, Proteintech), PSD95-specific, DLG4 polyclonal antibody (1:2000, 20,665-1-AP, Proteintech), and GAPDH antibody (1:50000, 60,004-1-Ig, Proteintech). The densities of the immunoblotting bands were quantified using ImageJ software (1.53q, National Institutes of Health, United States). All results were normalized against the average levels of the corresponding proteins detected in the normal chow diet (NCD) group, and subsequently expressed as relative densities of the respective bands.

### Determination of serum levels of parameters

Blood samples were collected and centrifuged at 4°C, 800 g for 15 min. The serum levels of total cholesterol (TC) and low-density lipoprotein cholesterol (LDL-C) were measured using a Chemistry Analyzer (BS-240vet, Mindray Bio-Medical Electronics, Shenzhen, China). To detect serum oxLDL level, a mouse oxLDL ELISA kit (EM0400, Wuhan Fine Biotech, Wuhan, China) was utilized following the manufacturer’s instructions. Mature serum insulin level was quantified utilizing a mouse ultrasensitive insulin ELISA kit (80-INSMSU-E01, ALPCO, Salem, NH, United States). Homeostasis model assessment-insulin resistance (HOMA-IR) was determined by HOMA2 calculator v2.2.3 (Diabetes trials unit, University of Oxford, Oxford, United Kingdom).

### MDA level measurement

The livers were isolated from the euthanized mice using ice-cold PBS. The tissues were homogenized and sonicated in a cold saline solution, followed by centrifugation at 2500 rpm for 15 min. The resulting supernatants were collected and mixed with the medium provided by the MDA kit (A003-1, Nanjing Jiancheng Bioengineering Institute, Nanjing, China). The mixtures were thereafter heated at 95°C for 50 min and subsequently centrifuged at 3500 rpm for 10 min. The supernatant was measured at 532 nm using a spectrophotometer, following the manufacturer’s instructions. The MDA level was presented as the nanomoles per gram of protein sample.

### Glutathione (GSH) level detection

Liver samples were collected and homogenized in the medium provided by total GSH (T-GSH)/GSSH kit (A061-1, Nanjing Jiancheng Bioengineering Institute). The homogenized samples were subsequently centrifuged at 3500 rpm for 15 min. The resulting supernatant was measured at 405 nm using a spectrophotometer, following the manufacturer’s instructions. The levels of T-GSH and GSSH were expressed as micromoles per gram of protein sample.

### Statistical analysis

Statistical analysis was conducted using Prism software (GraphPad Software Inc., La Jolla, CA, USA). Significance between the two groups was determined using an unpaired two-tailed Student’s *t* test. For making comparisons among multiple groups with two fixed factors, two-way analysis of variance (ANOVA) was performed. For making comparisons among multiple groups with one fixed factor, one-way ANOVA or Kruskal-Wallis test was carried out, depending on the normality test. The post-hoc tests were performed as indicated in the figure legends when the ANOVA or Kruskal-Wallis test was significant. *p* < 0.05 was indicative of statistical significance. Statistical source data and details can be found in the Supplementary material Data Sheet.

## Results

### Positive effects of probucol on cognitive abilities and social behaviors in HFD-fed mice

To investigate the potential of probucol in mitigating the adverse effects of HFD on cognitive abilities and social behaviors, C57BL/6 J mice were assigned to either the NCD group or the 60% HFD group (Supplementary Figure S1) for 8 weeks. Subsequently, HFD-fed mice were divided into two groups based on their body weight. One group received drinking water supplemented with 0.005% probucol (estimated to provide a dosage range of 10–25 mg/kg/day), while the other two groups maintained their original diet without any supplementary treatment. Consistent with previous studies (Yoshida et al., 2005; Kondo et al., 2006), probucol had no discernible effect on the consumption of food and water by mice (Supplementary Figure S2). After 12 weeks of probucol intervention, mice were subjected to a series of behavioral tests (Figure 1A). In the MWM test, untreated HFD-fed mice displayed increased latency to reach the hidden platform compared with NCD-fed mice during the 7-day navigation task (HFD to NCD, latency to platform, *t* = 5.655, *p* < 0.0001; area under the curve, *p* = 0.0002), indicating the reduced learning ability of mice. The latency to platform was significantly reduced in mice treated with probucol compared with untreated HFD-fed mice (HFD + probucol to HFD, latency to platform, *t* = 4.389, *p* < 0.0001; area under the curve, *p* = 0.0118), particularly to a level comparable to that of the NCD-fed mice (HFD + probucol to NCD, latency to platform, *t* = 1.295, *p* = 0.1957; area under the curve, *p* = 0.8444) (Figures 1B–D). Following the navigation task, mice underwent probe trials to evaluate their spatial memory ability. Notably, untreated HFD-fed mice exhibited significantly reduced time spent in the target quadrant (HFD to NCD, *q* = 4.371, *p* = 0.0072), fewer target-crossing events (HFD to NCD, *p* = 0.0165), and increased mean distance to the target (HFD to NCD, *q* = 4.22, *p* = 0.0099). Conversely, no significant distinctions were identified in these parameters when comparing NCD-fed mice with mice treated with probucol while on the HFD (HFD + probucol to NCD, time in target quadrant, *q* = 1.311, *p* = 0.6244; target-crossing events, *p* = 0.7894; mean distance to target, *q* = 1.31, *p* = 0.6250) (Figures 1E–H), indicating that the administration of probucol resulted in a moderate reversal of the impairments induced by HFD. These data suggest that probucol could antagonize the impairment of spatial reference memory by HFD. In contrast, neither the HFD nor the administration of probucol had any influences on the alternative triplet in Y maze test (Kruskal-Wallis statistic = 1.656, *p* = 0.4370), suggesting that spatial working memory remained unaffected by HFD (Supplementary Figure S3).

Social behaviors were also assessed using the three-chamber social approach task. Probucol-treated mice displayed a significant preference for interacting with stranger mice over exploring an empty cage (stranger to empty, time around cage, *t* = 3.356, *p* = 0.0060; distance around cage, *t* = 3.466, *p* = 0.0046), whereas untreated HFD-fed mice did not exhibit such a preference (stranger to empty, time around cage, *t* = 0.9367, *p* = 0.5898; distance around cage, *t* = 0.7959, *p* = 0.6807) (Figures 1I–K). Noteworthy, HFD did not impair social preference of mice to interact with stranger mice over a familiar one in the subsequent social preference test (stranger to familiar, time around cage, *t* = 5.23, *p* < 0.0001; distance around cage, *t* = 5.53, *p* < 0.0001) (Supplementary Figure S4). These findings indicate that probucol treatment could be protective against HFD-induced damage to spatial learning, memory and social interaction in mice.

### Probucol did not alleviate HFD-induced affective behaviors in mice

**Figure 2:**
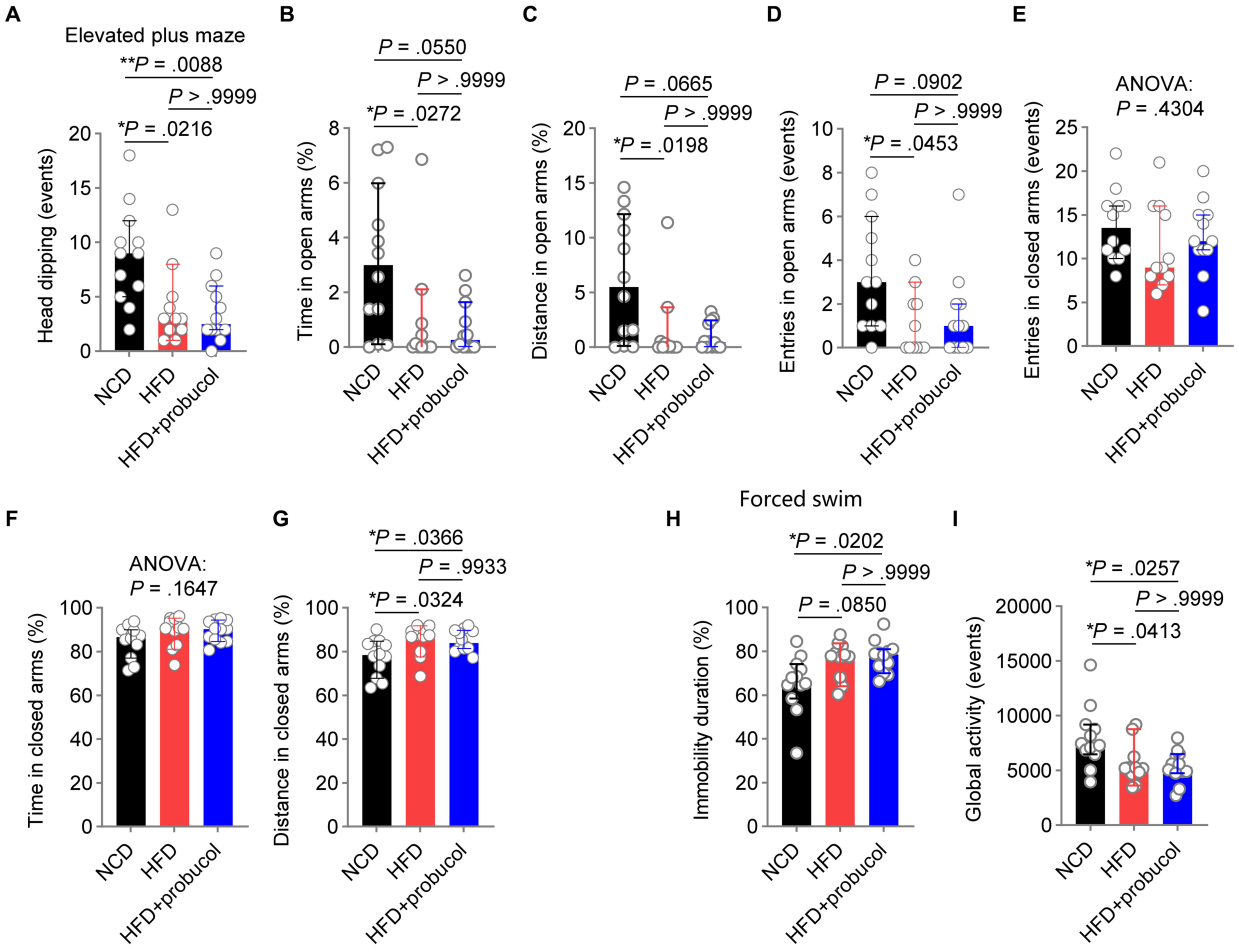
Probucol has no effect on anxiety and depression-like behaviors in HFD-fed mice. **(A–G)** Performance of mice in the elevated plus maze test. The data are expressed as individual values with median ± 95% CI (*n* = 11 or 12 mice for each group). Head dipping, Kruskal-Wallis test (*p* = 0.0044), followed by Dunn’s multiple comparisons test. Time in open arms, Kruskal-Wallis test (*p* = 0.0154), followed by Dunn’s multiple comparisons test. Distance in open arms, Kruskal-Wallis test (*p* = 0.0137), followed by Dunn’s multiple comparisons test. Entries in open arms, Kruskal-Wallis test (*p* = 0.0278), followed by Dunn’s multiple comparisons test. Entries in closed arms, Kruskal-Wallis test (*p* = 0.4304). Time in closed arms, ordinary one-way ANOVA (*p* = 0.1647). Distance in closed arms, ordinary one-way ANOVA (*p* = 0.0173), followed by Tukey’s multiple comparison. **(H,I)** Performance of mice in the forced swim test. The immobility duration and global activity are presented as individual values with median ± 95% CI (*n* = 11 or 12 mice for each group). Immobility duration, Kruskal-Wallis test (*p* = 0.0155), followed by Dunn’s multiple comparisons test. Global activity, Kruskal-Wallis test (*p* = 0.0127), followed by Dunn’s multiple comparisons test. *p* < 0.05 was indicative of statistical significance.

It has been reported that HFD could also induce anxiety and depression-like behaviors in mice. To determine whether probucol possesses anti-anxiety properties in HFD-fed mice, the EPM test was conducted. Both the untreated and probucol-treated HFD-fed mice exhibited significantly reduced head dipping (HFD to NCD, *p* = 0.0216; HFD + probucol to NCD, *p* = 0.0088), time spent on the open arms (HFD to NCD, *p* = 0.0272; HFD + probucol to NCD, *p* = 0.0550), distance traveled on the open arms (HFD to NCD, *p* = 0.0198; HFD + probucol to NCD, *p* = 0.0665), and entries in the open arms (HFD to NCD, *p* = 0.0453; HFD + probucol to NCD, *p* = 0.0902), as well as significantly increased distance traveled in the closed arms (HFD to NCD, *q* = 3.747, *p* = 0.0324; HFD + probucol to NCD, *q* = 3.671, *p* = 0.0366) compared with NCD-fed mice. There was no significant difference caused by HFD or probucol treatment in the entries in closed arms (ANOVA, *F* = 0.8657, *p* = 0.4304) and the time in closed arms (Kruskal-Wallis statistic = 3.607, *p* = 0.1647) (Figures 2A–G). These results suggested that anxiety level was similarly developed in both groups of mice fed with HFD. Additionally, a forced swim test was conducted to assess the potential effects of probucol on depression-like behaviors. Both untreated and probucol-treated HFD-fed mice exhibited moderately extended time of immobility (HFD to NCD, *p* = 0.0850; HFD + probucol to NCD, *p* = 0.0202) and significantly reduced global activity (HFD to NCD, *p* = 0.0413; HFD + probucol to NCD, *p* = 0.0257) compared to the NCD-fed mice (Figures 2H,I). These findings indicated that the beneficial effects of probucol were selective in targeting the cognitive performance affected by HFD, while did not influence affective behaviors.

### Probucol did not alleviate HFD-induced systemic metabolic disorders

**Figure 3:**
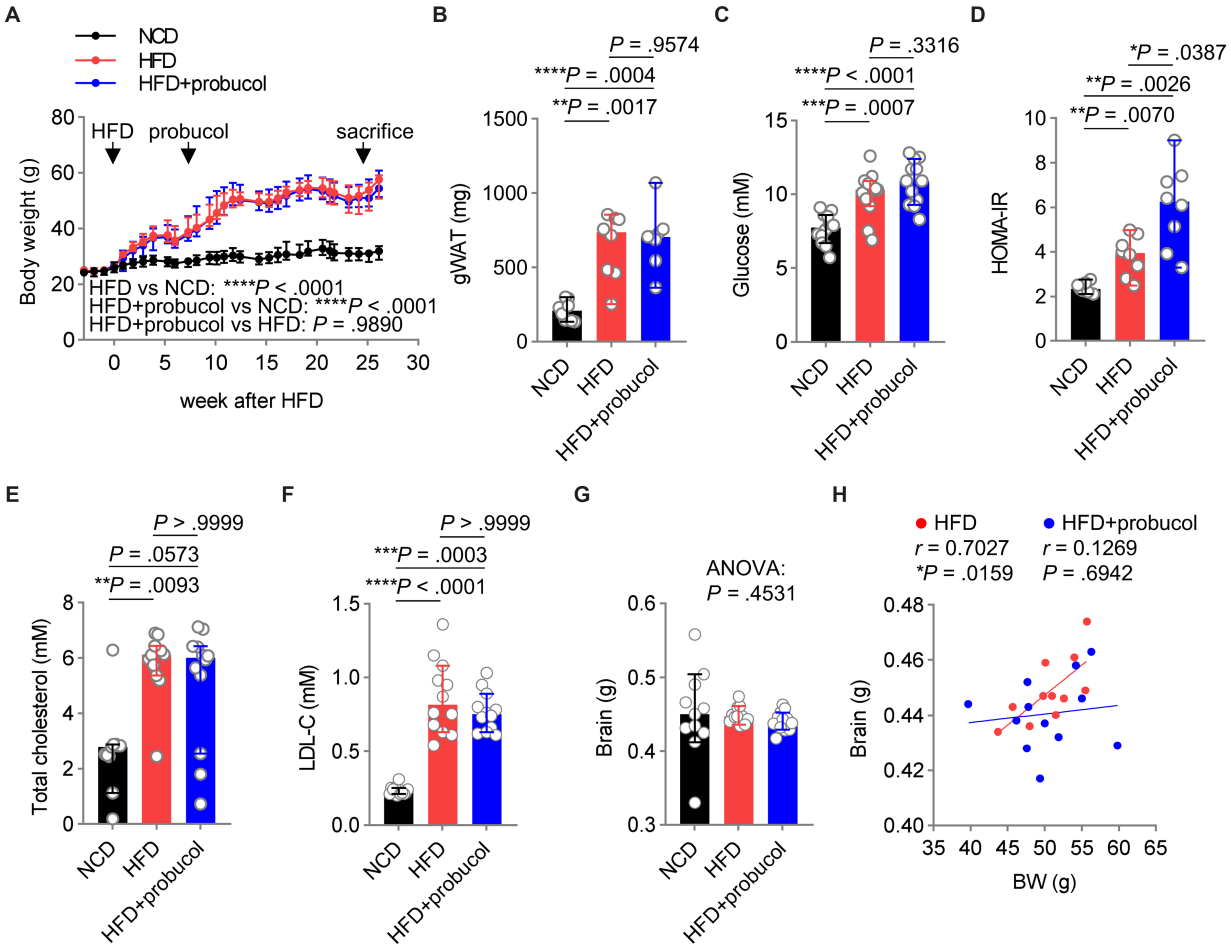
Probucol has no metabolic beneficial effects on HFD fed mice. **(A)** Probucol treatment has no effect on the body weight of mice. Data are presented as median ± 95% confidence interval (CI), and the slopes of the growth curves are compared by simply linear regression. **(B–F)** Probucol treatment has no effect on metabolic parameters of mice. The mass of gWAT and HOMA-IR of mice that received probucol for 5 weeks were analyzed (*n* = 7–8 mice for each group). The other parameters were analyzed for mice treated with probucol for 12 weeks (*n* = 12 mice for each group). Blood of mice was collected after 6 or 8 h fasting. Data are expressed as individual values with median ± 95% CI. The difference in gWAT mass between groups was compared by Brown-Forsythe ANOVA (*p* < 0.0001) followed by Tamhane’s T2 multiple comparisons test. Blood glucose, ordinary one-way ANOVA (*p* < 0.0001), followed by Tukey’s multiple comparisons test. HOMA-IR, Brown-Forsythe ANOVA (*p* = 0.0005), followed by Tamhane’s T2 multiple comparisons test. Total cholesterol, Kruskal-Wallis test (*p* = 0.0081) followed by Dunn’s multiple comparisons test. LDL-cholesterol, Kruskal-Wallis test (*p* < 0.0001) followed by Dunn’s multiple comparisons test. **(G)** Brain weights of mice. Brains dissected from mice were weighted and shown as individual values with median ± 95% CI (*n* = 11 or 12 mice for each group). Kruskal-Wallis test (*p* = 0.4531). **(H)** The correlations between body weights and the brain mass of HFD-fed mice and probucol treated mice. The degree of correlations was measured by Pearson’s correlation. *p* < 0.05 was indicative of statistical significance.

As dysregulated metabolism is considered as a primary cause of various comorbidities associated with HFD, including impaired cognitive functions, the effects of probucol on the metabolic profiles of these mice were assessed. Notably, body weight (HFD to NCD, *F* = 100.8, *p* < 0.0001) and the mass of gonadal white adipose tissue (gWAT) (HFD to NCD, *t* = 5.404, *p* = 0.0017) significantly increased in HFD-fed mice, which were not mitigated by probucol treatment (HFD + probucol to HFD, body weight, *F* = 0.0001934, *p* = 0.9890; gWAT, *t* = 0.4628, *p* = 0.9574) (Figures 3A,B). Moreover, probucol showed no impact on HFD-induced hyperglycemia (HFD to NCD, *q* = 5.845, *p* = 0.0007; HFD + probucol to HFD, *q* = 2.039, *p* = 0.3316). Instead, probucol treatment could exacerbate HFD-induced hyperinsulinemia (HFD to NCD, *t* = 4.132, *p* = 0.0070; HFD + probucol to HFD, *t* = 3.372, *p* = 0.0254) and systemic insulin resistance assessed by HOMA-IR (HFD to NCD, *t* = 4.367, *p* = 0.0070; HFD + probucol to HFD, *t* = 3.013, *p* = 0.0387) (Figures 3C,D and Supplementary Figure S5A). Furthermore, HFD-feeding significantly elevated serum levels of TC (HFD to NCD, *p* = 0.0093) and LDL-C (HFD to NCD, *p* < 0.0001), which were not reduced after administration of probucol (HFD + probucol to HFD, cholesterol, *p* > 0.9999; LDL-C, *p* > 0.9999) (Figures 3E,F). These results indicated that probucol did not exhibit a noticeable effect on alleviating HFD-induced metabolic disorders, suggesting that its beneficial effects could not be achieved through antagonizing the overall metabolic changes.

Reduced brain weight has been reported in mouse models of cognitive decline (Caccamo et al., 2017; Perepelkina et al., 2020; Winslow et al., 2021). In the present study, the organ weights of mice were determined, and it was revealed that neither HFD-feeding nor probucol treatment could significantly change brain weight (Kruskal-Wallis statistic = 1.583, *p* = 0.4531) (Figure 3G). However, a strongly positive correlation was identified between brain weight and body weight in untreated HFD-fed mice (*r* = 0.7027, *p* = 0.0159), which was not observed in the probucol-treated group (*r* = 0.1269, *p* = 0.6942) (Figure 3H). In contrast, robust positive correlations between body weight and other organs such as the kidney (HFD, *r* = 0.6842, *p* = 0.0202; HFD + probucol, *r* = 0.8895, *p* = 0.0001), liver (HFD, *r* = 0.8771, *p* = 0.0004; HFD + probucol, *r* = 0.9560, *p* < 0.0001) and spleen (HFD, *r* = 0.8329, *p* = 0.0028; HFD + probucol, *r* = 0.7737, *p* = 0.0031) were consistently found in both the untreated and probucol-treated mice (Supplementary Figures S5B–D). These data indicated that probucol could exert its effect particularly on the brain.

### The influence of probucol on oxidative stress in HFD-fed mice

**Figure 4:**
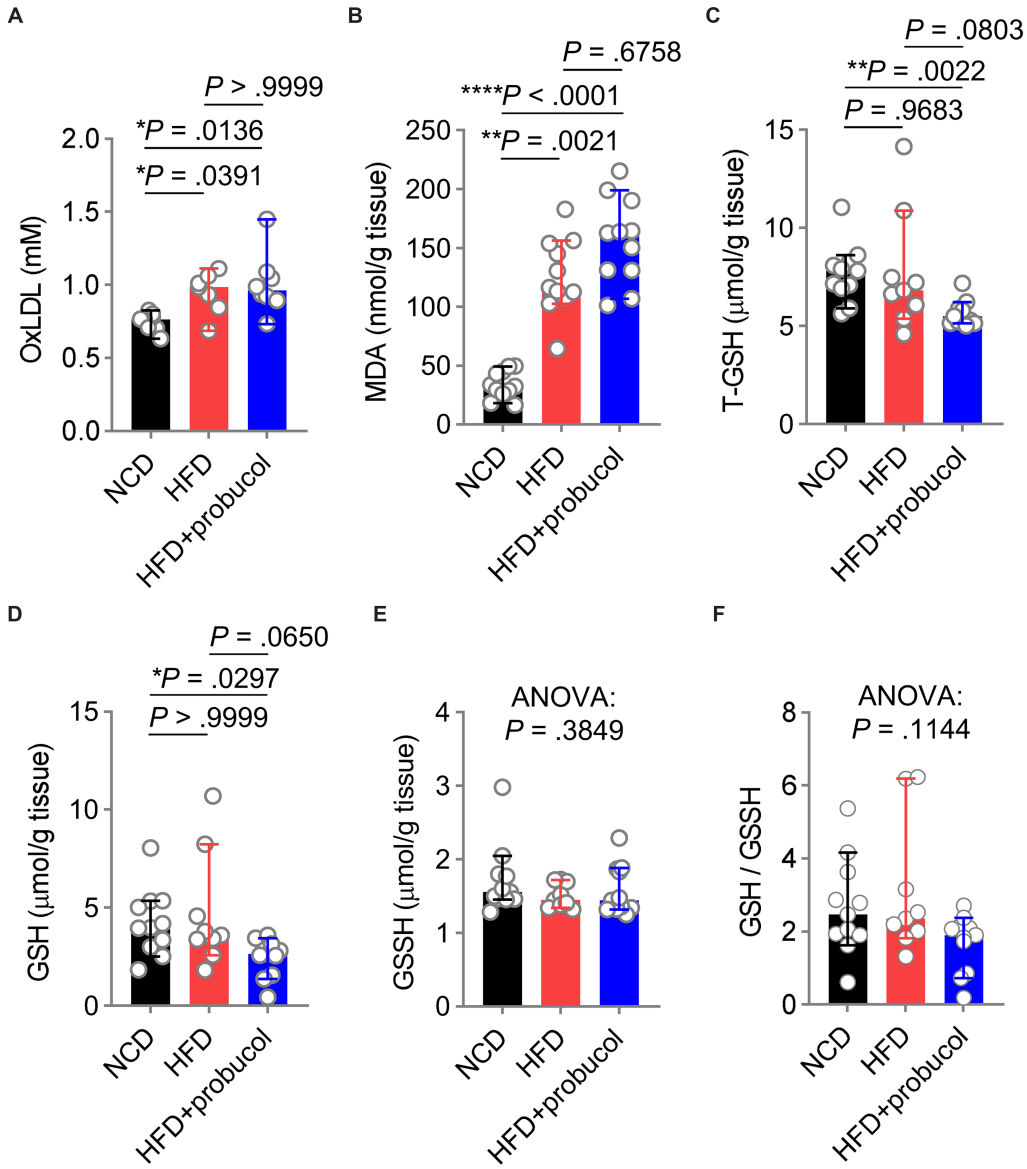
Influences of probucol feeding on mice redox status. **(A,B)** The levels of oxLDL **(A)** and MDA **(B)** in mice. Data are presented as individual values with median ± 95% CI (*n* = 7 or 8 mice per group for oxLDL, *n* = 11 mice per group for MDA). OxLDL, Kruskal-Wallis test (*p* = 0.0080), followed by Dunn’s multiple comparisons test. MDA, Kruskal-Wallis test (*p* < 0.0001), followed by Dunn’s multiple comparisons test. **(C–F)** The levels of T-GSH **(C)**, GSH **(D)**, GSSH **(E)**, and the GSH:GSSH ratio **(F)** in mice. Data are presented as individual values with median ± 95% CI (*n* = 9 or 11 mice for each group). T-GSH, Kruskal-Wallis test (*p* = 0.0026), followed by Dunn’s multiple comparisons test. GSH, Kruskal-Wallis test (*p* = 0.0171), followed by Dunn’s multiple comparisons test. GSSH, Kruskal-Wallis test (*p* = 0.3849). GSH/GSSH, Kruskal-Wallis test (*p* = 0.1144). *p* < 0.05 was indicative of statistical significance.

To explore the influences of probucol on alleviating HFD-induced oxidative stress, the level of oxLDL, a major target of probucol was measured. Notably, similar increases in oxLDL levels were found in both untreated and probucol-treated HFD-fed mice compared with NCD-fed mice (HFD to NCD, *p* = 0.0391; HFD + probucol to NCD, *p* = 0.0136; HFD + probucol to HFD, *p* > 0.9999) (Figure 4A). Furthermore, the level of MDA, an end product of lipid peroxidation and a major source of oxLDL modification, was similarly increased in the liver tissues of both the probucol-treated and untreated HFD-fed mice compared with NCD-fed mice (HFD to NCD, *p* = 0.0021; HFD + probucol to NCD, *p* < 0.0001; HFD + probucol to HFD, *p* = 0.6758) (Figure 4B).

Moreover, reduced GSH level, a crucial scavenger for ROS, was measured in these mice. Notably, T-GSH level was significantly reduced in probucol treated mice compared with both NCD-fed mice and untreated HFD-fed mice (HFD + probucol to NCD, *p* = 0.0022; HFD + probucol to HFD, *p* = 0.0803). This reduction was achieved by decreasing the reduced GSH level (HFD + probucol to NCD, *p* = 0.0297; HFD + probucol to HFD, *p* = 0.0650), while the oxidative glutathione (GSSH) level remained unchanged (Kruskal-Wallis statistic = 1.909, *p* = 0.3849). As a result, the GSH:GSSH ratio was also moderately reduced in probucol-treated mice (Kruskal-Wallis statistic = 4.335, *p* = 0.1144) (Figures 4C–F). These findings suggested that probucol did not act to counteract the systemic oxidative stress induced by an HFD.

### Probucol counteracts the impact of HFD by antagonizing hippocampal insulin resistance and differentially regulating radical species

**Figure 5:**
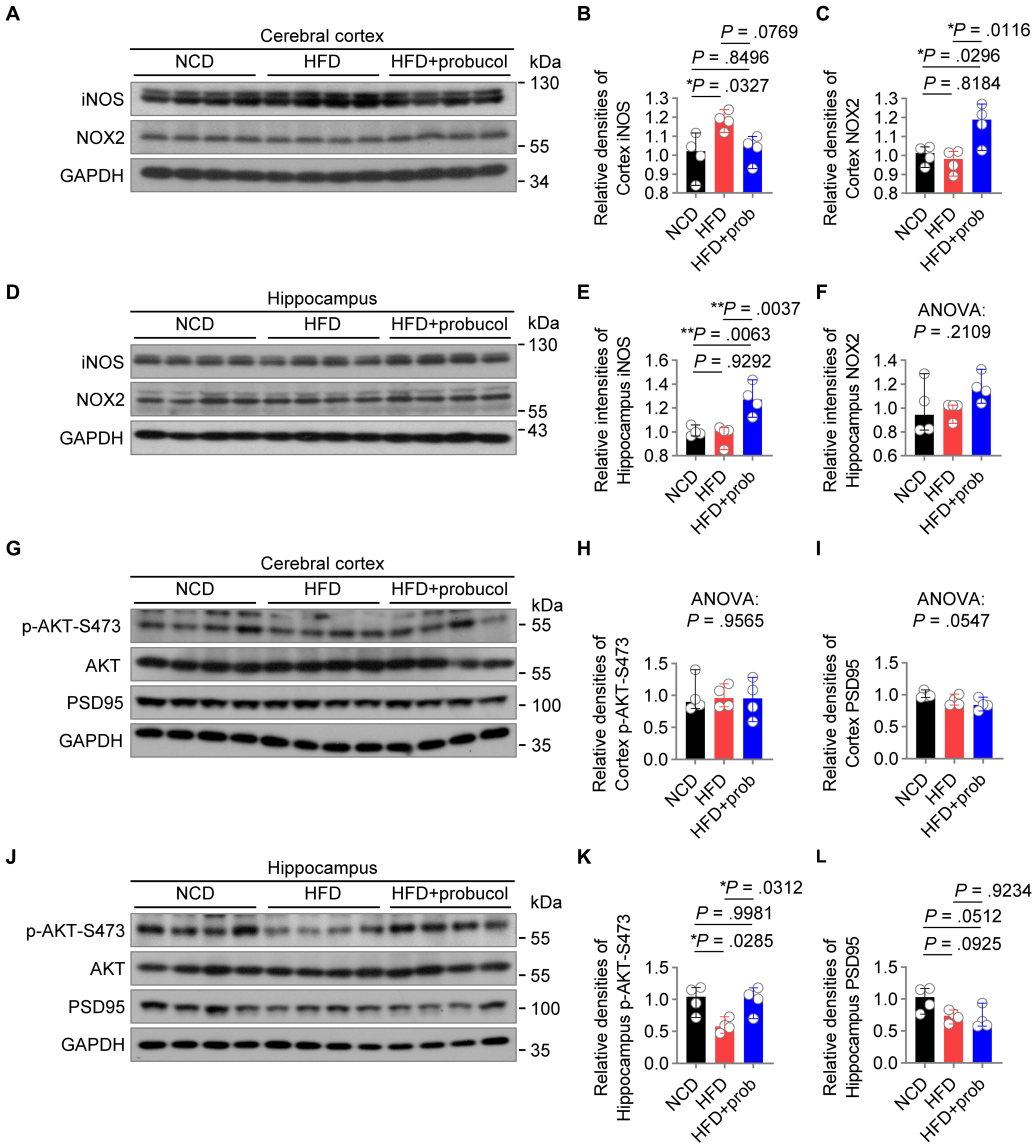
Probucol alleviates hippocampal insulin resistance and differentially regulates radical species in cerebral cortex of mice. **(A–F)** Western blot analysis of lysates of cerebral cortex and hippocampus from male mice with or without probucol administration for 12 weeks. The levels of proteins were quantified in the left (*n* = 4 mice per group). Cortex iNOS, ordinary one-way ANOVA (*p* = 0.0296), followed by Tukey’s multiple comparisons test. Cortex NOX2, ordinary one-way ANOVA (*p* = 0.0099), followed by Tukey’s multiple comparisons test. Hippocampal iNOS, ordinary one-way ANOVA (*p* = 0.0025), followed by Tukey’s multiple comparisons test. Hippocampal NOX2, ordinary one-way ANOVA (*p* = 0.2109). **(G–L)** Western blot analysis of AKT phosphorylation and PSD95 in the brain extracts of male mice with or without probucol administration for 5 weeks (*n* = 4 mice per group). Cortex p-AKT, ordinary one-way ANOVA (*p* = 0.9565). Cortex PSD95, ordinary one-way ANOVA (*p* = 0.0547). Hippocampus p-AKT, ordinary one-way ANOVA (*p* = 0.0179), followed by Tukey’s multiple comparisons test. Hippocampus PSD95, ordinary one-way ANOVA (*p* = 0.0435), followed by Tukey’s multiple comparisons test. *p* < 0.05 was indicative of statistical significance.

In order to examine the molecular mechanisms underlying the beneficial effects of probucol on cognitive performance, the levels of candidate proteins mediating HFD-induced inflammation and oxidative stress in the brain were analyzed. In the cortex, HFD-feeding significantly increased iNOS level (HFD to NCD, *q* = 4.333, *p* = 0.0327), which is responsible for neurodegenerative changes in the cortex (Madrigal et al., 2001). Probucol treatment counteracted the effects of HFD on the induction of iNOS in the cortex, restoring its level to that found in the cortex of NCD-fed mice (HFD + probucol to NCD, *q* = 0.7764, *p* = 0.8496) (Figures 5A,B). However, in the hippocampus, while HFD had no effect on iNOS level (HFD to NCD, *q* = 0.5182, *p* = 0.9292), probucol treatment increased iNOS level (HFD + probucol to NCD, *q* = 5.874, *p* = 0.0063; HFD + probucol to HFD, *q* = 6.392, *p* = 0.0037) (Figures 5D,E). The level of NOX2, a major source of superoxide in the brain, was significantly elevated in the cortex (HFD + probucol to NCD, *q* = 4.426, *p* = 0.0296; HFD + probucol to HFD, *q* = 5.288, *p* = 0.0116) and moderately accumulated in the hippocampus of probucol-treated mice (ANOVA, *F* = 1.859, *p* = 0.2109) compared with both NCD-fed mice and untreated HFD-fed mice (Figures 5A,C,D,F). Given the crucial roles of ROS in learning, memory, and brain plasticity (Kishida et al., 2006; Dickinson et al., 2011), probucol may combat the detrimental effects of HFD by upregulating NOX2 levels. These data indicated that probucol could differentially regulate the machineries for nitric oxide and superoxide in the cortex.

Brain insulin resistance may play a notable role in mediating the effect of high-nutrition on neuroinflammation and redox homeostasis (Spinelli et al., 2017; Zhang et al., 2022), leading to synaptic damage and cognition deficits (Xie et al., 2021). Hence, the phosphorylation of AKT in the brain was examined, and its impairment was indicative of insulin resistance. HFD-feeding has led to a reduction in p-AKT-S473 levels specifically in the hippocampus (HFD to NCD, *q* = 4.459, *p* = 0.0285) of mice. Administration of probucol restored AKT phosphorylation (HFD + probucol to NCD, *q* = 0.08332, *p* = 0.9981; HFD + probucol to HFD, *q* = 4.375, *p* = 0.0312). Comparatively, neither the HFD nor probucol could exert any notable influences on p-AKT-Ser473 level in the cerebral cortex (ANOVA, *F* = 0.04473, *p* = 0.9565) (Figures 5G,H,J,K). Of note, HFD-feeding also resulted in a mild reduction in the level of PSD95, a synaptic marker, in the hippocampus (HFD to NCD, *q* = 3.387, *p* = 0.0925), whereas probucol treatment did not restore PSD95 level (HFD + probucol to NCD, *q* = 3.928, *p* = 0.0512; HFD + probucol to HFD, *q* = 0.5403, *p* = 0.9234). In addition, both the HFD-feeding and probucol treatment did not significantly affect PSD95 level in the cortex (ANOVA, *F* = 4.082, *p* = 0.0547) (Figures 5G,I,J,L). These findings indicated that probucol administration could selectively alleviate insulin resistance in the hippocampus of HFD-fed mice.

## Discussion

HFD is regarded as an important factor not only for the development of metabolic disorders but also for CNS abnormalities (Custers and Kiliaan, 2022). In the present study, the effects of probucol on HFD-fed mice were systematically examined in terms of cognitive abilities, social behaviors and mood-related behaviors, metabolism and redox status. Probucol demonstrated notable benefits in counteracting the decline in spatial learning and memory induced by an HFD, while also enhancing sociability (Figure 1). Furthermore, it was revealed that various cognitive tasks exhibited disparate susceptibility to the effects of HFD or probucol. For instance, HFD-treated mice displayed normal performance in the Y maze spontaneous alternation test (Supplementary Figure S3), while probucol did not exhibit any discernible effects in alleviating depression-like behaviors in mice (Figure 2). This divergence aligns with the distinct influences of HFD or probucol on the molecular markers in the hippocampus and cerebral cortex (Figure 5), suggesting the complex coordination and interaction among different brain regions during various cognitive tasks. The commencement of the first phase II trial of probucol on cognitive function in Alzheimer’s disease (Lam et al., 2022) may provide valuable clinical insights into the effects of probucol on cognition upon its completion.

Numerous clinical studies have reported the modest cholesterol-lowering effects of probucol in patients with hypercholesterolemia (Walldius et al., 1994; Arai et al., 2022), while results of studies conducted on mice have demonstrated varied systemic metabolic effects (Zhang et al., 1997; Bird et al., 1998; Guttapadu et al., 2023). In the present study, probucol treatment did not counteract HFD-induced systemic metabolic disturbances (Figures 3, 4 and Supplementary Figure S5). These discrepancies in the systemic metabolic effects of probucol may be attributed to factors such as drug dosage, administration methods, and the distinctive lipid profiles between humans and mice. In clinical practice, a higher dosage of 1 g of probucol is typically recommended due to the limited 6% bioavailability through the common oral route. However, the estimated equivalent dose for mice is approximately 350–700 mg/kg/daily, which is remarkably higher than the dose utilized in this study (10–25 mg/kg/daily in drinking water). Other studies have reported that the addition of probucol to the diet had no discernible impact on liver weight or liver cholesterol level in rats (Barnhart et al., 1970). Additionally, when administered orally, probucol did not exhibit any additional effects on serum total cholesterol, triacylglycerol, and oxLDL levels in apolipoprotein E-deficient mice treated with atorvastatin and fed with HFD (Guo et al., 2019). Despite intraperitoneal injection of probucol at doses of 10 or 20 mg/kg/day for 6 weeks into Kunming mice, both normal and D-galactose-treated mice, no notable effects on their body weight were found (Huang J. L. et al., 2019). Furthermore, it is vital to recognize the contrasting plasma lipid profiles between humans and mice (Yin et al., 2012), as well as variations among different mouse models. For instance, LDL functions as the primary cholesterol carrier in human plasma, and the involvement of LDL is essential for mediating the cholesterol-lowering effects of probucol (Urien et al., 1984; O'Leary et al., 1996). In contrast, the majority of cholesterol in mice, regardless of whether they were on a NCD or a HFD, is associated with HDL. In the present study, HFD-fed mice were not transitioned to an NCD during probucol administration. Comparably, patients diagnosed with hypercholesterolemia typically modify their diet to a healthier one and implement additional lifestyle changes. These factors could potentially diminish the sensitivity of mice to probucol treatment with regards to systemic metabolic parameters. Given the substantial discovery of small-molecule drugs as major effectors of the composition of microbiota (Lindell et al., 2022), it is essential to further examine the influences exerted by unabsorbed probucol on the gut microbiota. This exploration may unravel the underlying mechanisms by which probucol affects cognitive function.

The positive correlation between brain weight and body weight in untreated HFD-fed mice indicated that neuronal cell hypertrophy or hyperplasia could be detrimental, and destruction of such correlation might contribute to the improvement of CNS function by probucol (Figure 3). In addition, the effects on the cerebral cortex and hippocampus induced by either HFD or probucol did not exhibit correlation across various parameters, including redox and neuroinflammatory markers, and AKT phosphorylation (Figure 5). Such disparities are reminiscent of findings in certain neurological disorders, where inverse correlations between cortical and subcortical regions in size and metabolic activities have been documented (Zhao et al., 2021; Liu et al., 2023). The bidirectional interaction between the hippocampus and cortex, along with the distinct cellular composition of these two structures, may underlie these differences, warranting further investigation.

The relevance of oxidative stress in obesity-associated comorbidities has been frequently studied (Furukawa et al., 2004; Gentile et al., 2018). It is broadly accepted that overproduction of free radicals and related inflammatory markers is detrimental, although these factors may be essential for normal physiological responses under specific stressed conditions such as cold-induced brown adipocyte thermogenesis (Chouchani et al., 2017). However, contrary to the potential antioxidant properties of probucol in severely diseased models, the findings of this study revealed an increase in systemic and regional oxidative stress in probucol-treated mice fed with HFD (Figure 4). Moreover, probucol could upregulate lipid peroxidation in erythrocyte and plasma in mice and macaques (Herbas et al., 2015; Shichiri et al., 2019), elevate serum NO level in Sprague Dawley rats (Jiang et al., 2002), and enhance NO bioactivity in aortic rings in rabbits (Lau et al., 2003). This is noteworthy, especially considering the role of iNOS in adult neurogenesis (Fernandes et al., 2021). Regarding the low LDL-C plasma level in mice compared with that in humans (Yin et al., 2012) and the reliance on incorporation into the LDL particles for provoking the antioxidant effect of probucol (Urien et al., 1984; O'Leary et al., 1996), it is essential to determine whether the potential antioxidant effect of probucol in patients with significantly elevated LDL-C levels, including those with familial hypercholesterolemia, is consistently replicated in specific mouse models. A growing body of evidence suggested that antioxidant effects may not always be the primary determinant. For instance, the metabolic/antioxidant function and the CNS effects of probucol were inconsistently found in animal models (Moreira et al., 2013; Huang J. L. et al., 2019). Conversely, beta-carotene, despite lacking an effect on LDL oxidation, has demonstrated efficacy in preventing lesion formation to a similar extent as probucol in cholesterol-fed rabbits (Shaish et al., 1995).

In conclusion, the present study revealed the potential of probucol in counteracting HFD-induced cognitive decline without imparting systemic metabolic benefits for reducing oxidative stresses. These findings call for a reconsideration of probucol’s mechanisms of action, as well as the significance of altered metabolic profiles and free radicals in brain function. Moreover, the findings underscore the importance to reassess the roles of metabolic shifts, redox homeostasis and inflammation in the development of diet-induced cognitive deficits.

## Data availability statement

The original contributions presented in the study are included in the article/Supplementary material, further inquiries can be directed to the corresponding authors.

## Ethics statement

The animal study was approved by the Institutional Animal Care and Use Committee at Xiamen University. The study was conducted in accordance with the local legislation and institutional requirements.

## Author contributions

H-MW: Data curation, Formal analysis, Investigation, Methodology, Validation, Visualization, Writing – original draft, Writing – review & editing. YY: Data curation, Formal analysis, Investigation, Methodology, Validation, Visualization, Writing – original draft, Writing – review & editing. N-JH: Data curation, Formal analysis, Investigation, Methodology, Writing – original draft. L-PF: Investigation, Methodology, Resources, Writing – original draft. Y-YD: Data curation, Formal analysis, Investigation, Methodology, Validation, Writing – review & editing. K-TH: Data curation, Formal analysis, Investigation, Methodology, Validation, Writing – review & editing. T-YT: Investigation, Writing – original draft. LL: Writing – original draft, Investigation. YX: Formal analysis, Writing – original draft. D-TL: Investigation, Methodology, Writing – original draft. Z-XC: Investigation, Writing – original draft, Writing – review & editing. X-YN: Investigation, Writing – review & editing. X-YR: Investigation, Writing – original draft. Z-HY: Investigation, Writing – original draft. H-YQ: Investigation, Writing – review & editing. J-ZC: Investigation, Writing – original draft. XH: Methodology, Writing – original draft. CZ: Methodology, Writing – original draft. XY: Methodology, Writing – original draft. CW: Resources, Writing – original draft. YH: Resources, Writing – original draft. WH: Project administration, Writing – original draft, Writing – review & editing. Y-HZ: Conceptualization, Funding acquisition, Project administration, Supervision, Writing – original draft, Writing – review & editing, Resources. S-YL: Conceptualization, Data curation, Formal analysis, Funding acquisition, Methodology, Project administration, Supervision, Validation, Visualization, Writing – original draft, Writing – review & editing. Y-XS: Writing – review & editing, Resource.
