# Supplementary material for: Probucol mitigates high-fat diet-induced cognitive and social impairments by regulating brain redox and insulin resistance
Source: Front Neurosci. 2024 Apr 23;18:1368552. doi: 10.3389/fnins.2024.1368552 (PMC11074470; doi:10.3389/fnins.2024.1368552)
Supplement: Supplementary file 2 [file Image_1.pdf]

	Normal chow diet		High-fat diet	
	g %	kcal %	g %	kcal %
Protein	19.2	20	26.2	20
Carbohydrate	67.3	70	26.3	20
Fat	4.3	10	34.9	60
Total				100
kcal/gram	3.85		5.24	
Ingredient	gram	kcal	gram	kcal
Casein, 80 mesh	200	800	200	800
L-Cystine	3	12	3	12
Cornstarch	315	1260	0	0
Maltodextrin 10	35	140	125	500
Sucrose	350	1400	68.8	275.2
Cellulose, BW200	50	0	50	0
Soybean Oil	25	225	25	225
Lard*	20	180	245	2205
Mineral Mix, S10026	10	0	10	0
Dicalcium Phosphate	13	0	13	0
Calcium Carbonate	5.5	0	5.5	0
Potassium Citrate, 1 H_2O	16.5	0	16.5	0
Vitamin Mix, V10001	10	40	10	40
Choline Bitartrate	2	0	2	0
FD and C Dye	0	0	0.05	0
Total	1055.05	4057	773.85	4057

*Estimated Lard Cholesterol: 300.8 mg/kg high-fat diet

Supplementary Figure S1
Diet formulas.

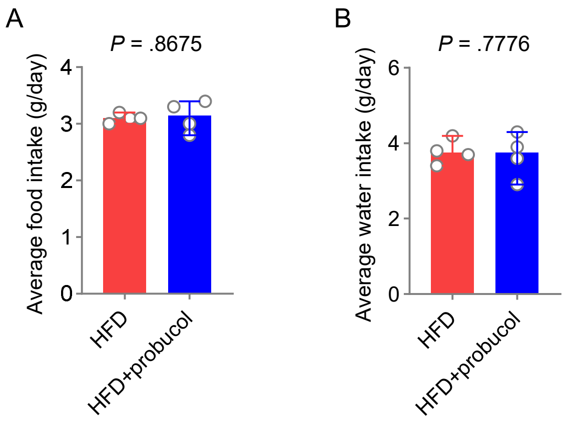

Supplementary Figure S2

Probucol did not significantly affect the consumption of food or water by mice. **(A, B)** After 12 weeks of probucol treatment, the intake of food **(A)** and water **(B)** in HFD-fed mice was recorded for each cage. The average daily consumption of food and water per mouse was calculated and presented as individual values with median ± 95% CI (two cages and two days for each group). Two-tailed unpaired Student's t test was used (food, t = 0.1741, p = 0.8675; water, t = 0.2954, p = 0.7776). $p <$ 0.05 was indicative of statistical significance.

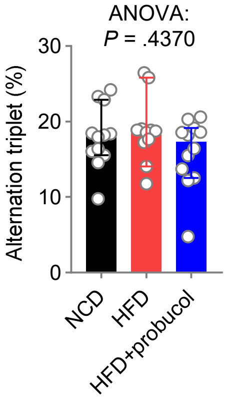

Supplementary Figure S3
HFD-feeding and probucol treatment did not display any discernible impact on the Y maze performance of mice.

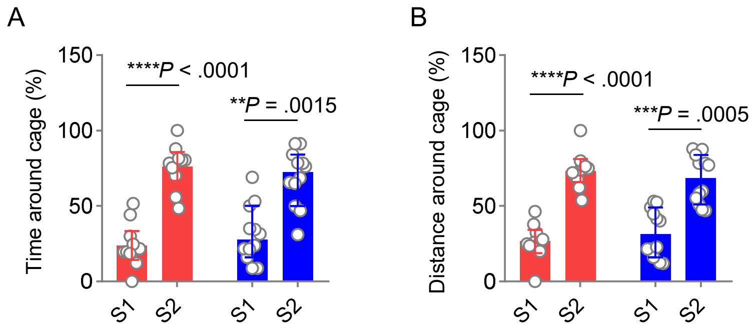

Supplementary Figure S4

HFD did not impair social preference of the mice for a stranger mouse over a familiar one. **(A, B)** Social preference test was performed following sociability test using the three-chamber social approach task. S1 and S2 indicate the cage with a familiar mouse introduced in the preceding social interaction phase and a new stranger mouse introduced in this social preference phase, respectively. The percentages of time spent around the cage **(A)** and the percentages of distance around the cage **(B)** are shown as individual values with median ± 95% CI (n = 11 or 12 mice for each group). Time around cage, two-way RM ANOVA (cage, $p < 0.0001$; group, $p = 0.5034$), followed by Sidak's multiple comparisons test. Distance around cage, two-way RM ANOVA (cage, $p < 0.0001$; group, $p > 0.9999$), followed by Sidak's multiple comparisons test. $p < 0.05$ was indicative of statistical significance.

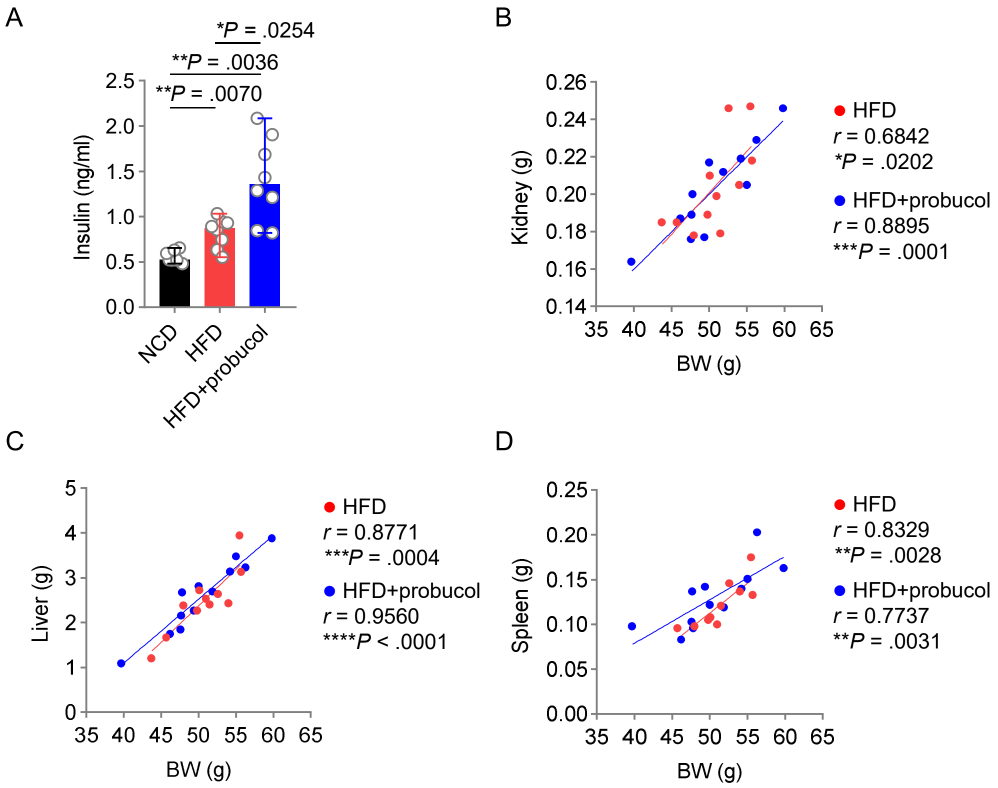

Supplementary Figure S5

Probucol exhibited no effect on the metabolic parameters of HFD-fed mice. **(A)** Serum insulin level of mice. Blood samples of mice were collected after 6-h fasting. Data are expressed as individual values with median ± 95% CI (n = 7-8 mice for each group). The difference in serum insulin level among groups was compared by Brown-Forsythe ANOVA (p = 0.0007), followed by Tamhane's T2 multiple comparisons test. **(B-D)** The correlations between body weights and the weights of kidney, liver and spleen of the HFD-fed mice and probucol treated mice. The degree of correlations was measured by Pearson r correlation. $p < 0.05$ was indicative of statistical significance.

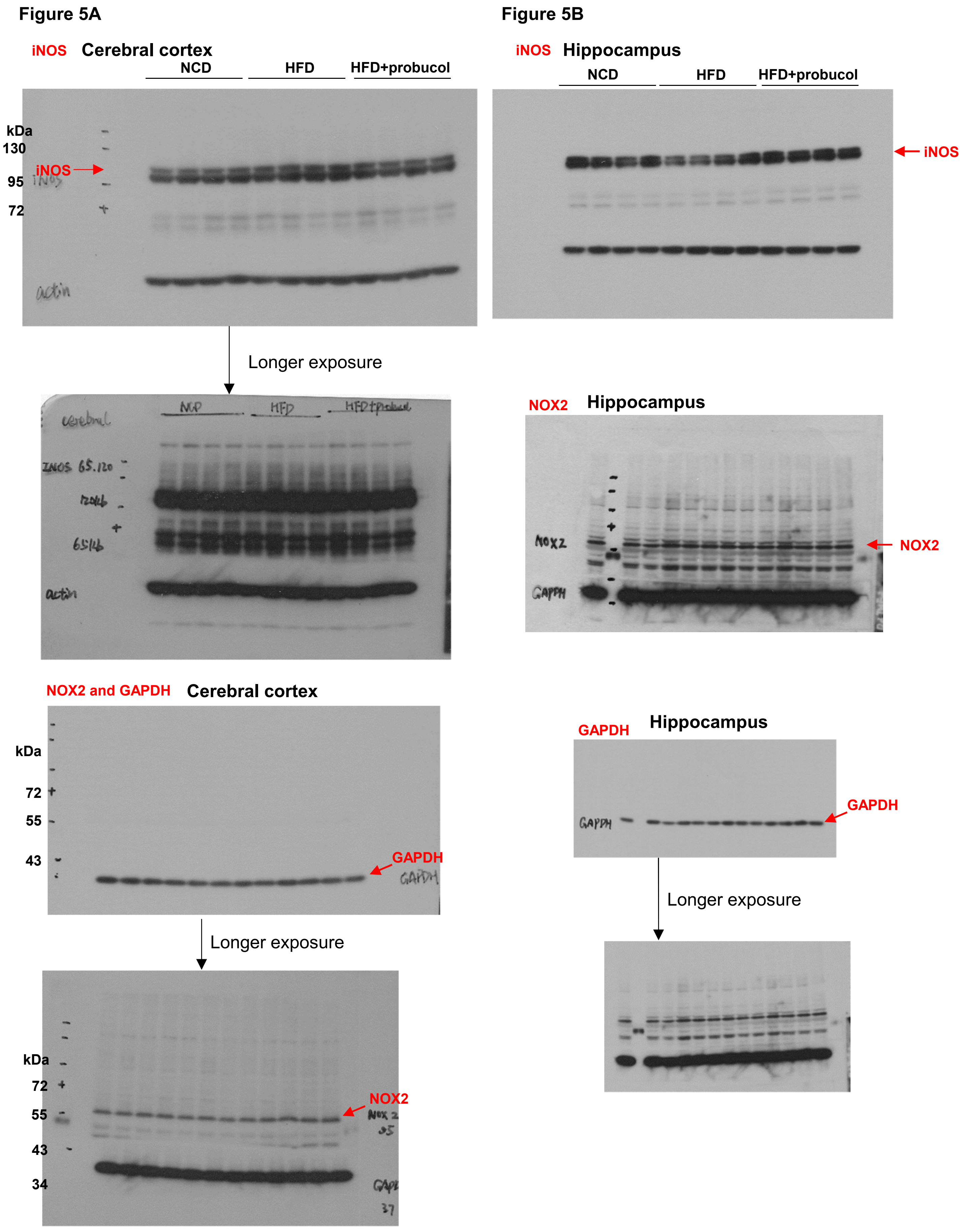

Supplementary Figure S6
Raw blots for Figure 5, A and D.

Figure 5, C and D

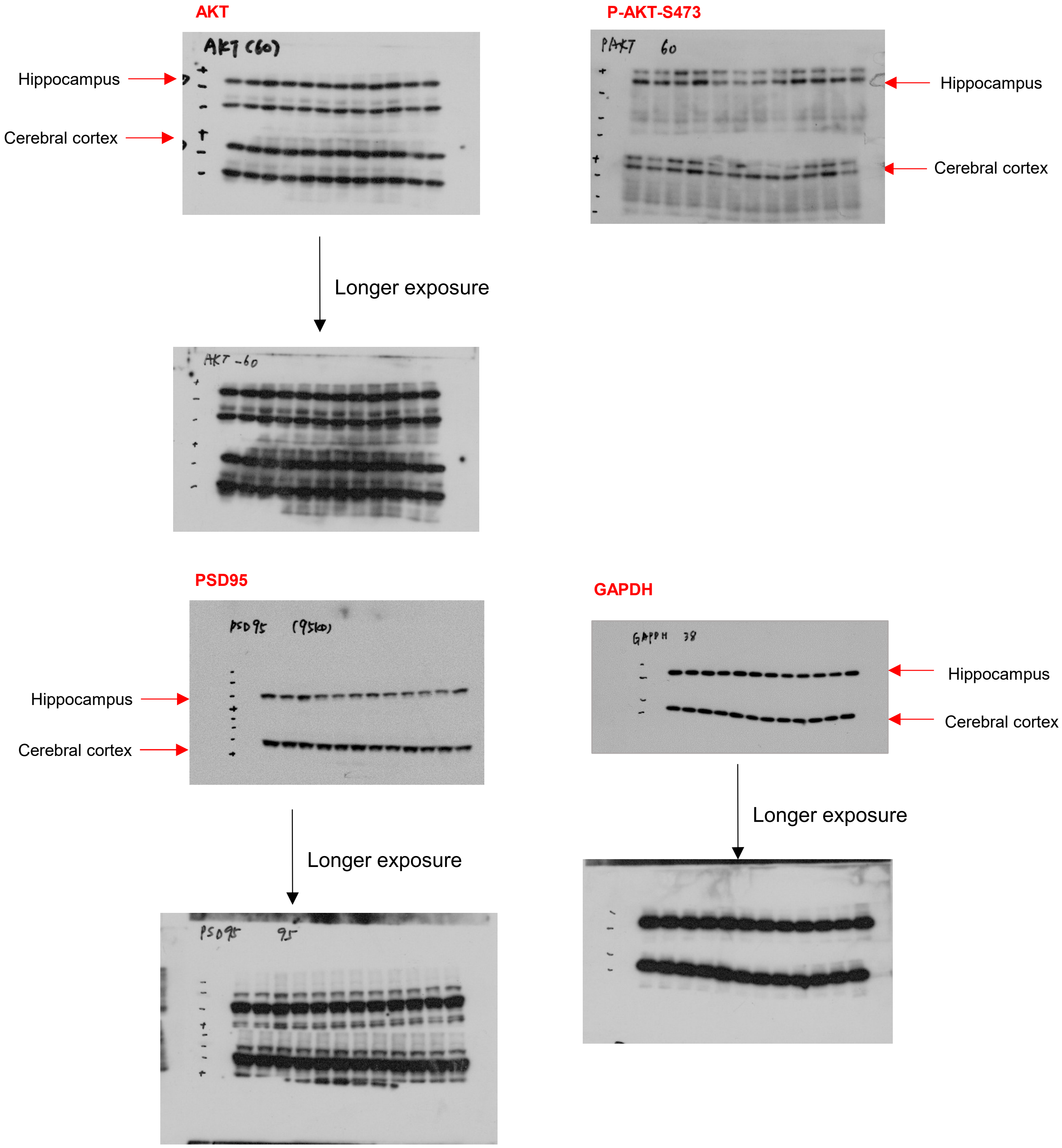

Supplementary Figure S7
Raw blots for Figure 5, G and J.
